# Frontonasal dysmorphology in bipolar disorder by 3D laser surface imaging and geometric morphometrics: Comparisons with schizophrenia

**DOI:** 10.1016/j.schres.2010.05.001

**Published:** 2010-09

**Authors:** Robin J. Hennessy, Patrizia A. Baldwin, David J. Browne, Anthony Kinsella, John L. Waddington

**Affiliations:** a Molecular and Cellular Therapeutics, Royal College of Surgeons in Ireland, St. Stephen's Green, Dublin 2, Ireland; b Cavan-Monaghan Mental Health Service, St. Davnet's Hospital, Monaghan, Ireland

**Keywords:** Bipolar disorder, Schizophrenia, Development, Dysmorphology, Craniofacies, Laser surface imaging, Geometric morphometrics

## Abstract

Any developmental relationship between bipolar disorder and schizophrenia engenders continuing debate. As the brain and face emerge in embryological intimacy, brain dysmorphogenesis is accompanied by facial dysmorphogenesis. 3D laser surface imaging was used to capture the facial surface of 13 male and 14 female patients with bipolar disorder in comparison with 61 male and 75 female control subjects and with 37 male and 32 female patients with schizophrenia. Surface images were analysed using geometric morphometrics and 3D visualisations to identify domains of facial shape that distinguish bipolar patients from controls and bipolar patients from those with schizophrenia. Both male and female bipolar patients evidenced significant facial dysmorphology: common to male and female patients was overall facial widening, increased width of nose, narrowing of mouth and upward displacement of the chin; dysmorphology differed between male and female patients for nose length, lip thickness and tragion height. There were few morphological differences in comparison with schizophrenia patients. That dysmorphology of the frontonasal prominences and related facial regions in bipolar disorder is more similar to than different from that found in schizophrenia indicates some common dysmorphogenesis. Bipolar disorder and schizophrenia might reflect similar insult(s) acting over slightly differing time-frames or slightly differing insult(s) acting over a similar time-frame.

## 1 Introduction

Few debates in psychiatry have the endurance of that surrounding the “Kraepelinian dichotomy”: are schizophrenia and bipolar disorder (i) distinct entities with separate underlying disease processes or (ii) positions along a continuum of pathobiology characterised by dimensions of psychopathology (Jablensky, 1999; Maier et al., 2006; Ivleva et al., 2008; van Os and Kapur, 2009)? This challenge continues to receive investigation at the levels of epidemiology (Mortensen et al., 2003; Baldwin et al., 2005; Hippisley-Cox et al., 2007), molecular genetics (Moskvina et al., 2009; O'Donovan et al., 2009) structural brain pathology (McDonald et al., 2005; Arnone et al., 2009; Fornito et al., 2009), neurological signs (Whitty et al., 2006), neurophysiology (Thaker, 2008) and cognitive dysfunction (Krabbendam et al., 2005; Schretlen et al., 2007; Hill et al., 2008).

In relation to the origins of psychosis, much theorising indicates an important role for early disturbance in brain development (Waddington et al., 1999a; McGrath et al., 2003; Rapoport et al., 2005; van Os and Kapur, 2009). However, understanding of the biological basis of this process is limited for schizophrenia and weaker still for bipolar disorder (Dutta et al., 2007). While recent studies have compared the developmental trajectories of schizophrenia and bipolar disorder, for example in terms of premorbid functioning (Zammit et al., 2004; Tiihonen et al., 2005; Rietschel et al., 2009), this challenge would be advanced by an index of early developmental disturbance that could be accessed directly in living patients to inform incisively on the nature of the underlying abnormality.

Slight anatomical malformations of body regions sharing the ectodermal origins of the brain are referred to as minor physical anomalies and indicate adverse events acting over the first or early second trimester (Waldrop et al., 1968; Smith, 1988). While they occur to consistent excess in schizophrenia (Waddington et al., 1999a; McNeil et al., 2000; Weinberg et al., 2007; Compton and Walker, 2009), they have received considerably less systematic study in bipolar disorder (Lloyd et al., 2007; Tenyi et al., 2009) and constitute a non-specific, qualitative indicator of early biological adversity. More incisively, distinct facial dysmorphologies in disorders of early brain development reflect the embryological intimacy with which the anterior brain and face evolve over early fetal life (Diewert et al., 1993; Kjaer, 1995; Waddington et al., 1999a, b; Schneider et al., 2001; Hennessy et al., 2004, 2005; Marcucio et al., 2005; Aoto et al., 2009). While anthropometric studies by ourselves (Lane et al., 1997; Hennessy et al., 2004) and others (McGrath et al., 2002; Donovan-Lepore et al., 2006) have indicated subtle facial dysmorphology in schizophrenia, this has yet to receive systematic study in bipolar disorder.

3D digitisation technologies now allow facial surfaces to be recorded in their entirety, while developments in geometric morphometrics now allow 3D analysis, both visual and statistical, of those surfaces (Hennessy et al., 2002, 2005; Hammond et al., 2004, 2005). As the developmental biology of facial morphogenesis is considerably better understood than is brain morphogenesis, resolution of the topography of facial dysmorphology in disorders of early brain development may lead to increased understanding of brain dysmorphogenesis. Thus, we have recently applied portable, hand-held 3D laser surface imaging and geometric morphometrics to identify in schizophrenia a topography of frontonasal and associated dysmorphologies (Hennessy et al., 2007).

In this report we describe the application of these same 3D laser surface imaging and geometric morphometric methods to investigate facial dysmorphology in patients with bipolar disorder and determine the extent to which such dysmorphology is similar to or different from that evident in patients with schizophrenia.

## 2 Materials and methods

### 2.1 Participants

Approval for this study was obtained from the Research Ethics Committee of the North Eastern Health Board; each subject gave written, informed consent to his/her participation. All procedures were identical to those detailed for our preceding study in schizophrenia (Hennessy et al, 2007). Thus, patients under the age of 65 were drawn from attendees of Cavan-Monaghan Mental Health Service, which applies a home-based, community model of care. Each satisfied DSM-IV criteria (American Psychiatric Association, 1994) for bipolar disorder, with or without psychotic features, and with exclusion of schizoaffective disorder, on the basis of clinical interview and case note review, as described previously (Scully et al., 2004; Baldwin et al., 2005); patients were receiving conventional treatment with mood stabilisers, supplemented as necessary by antipsychotic and antidepressant medication. Control subjects under the age of 65 were drawn from individual and community group volunteers in Cavan-Monaghan; on semi-structured interview with the same psychiatrist who assessed patients, those individuals giving a personal or family history of psychotic illness or suicide in a first-degree relative were excluded. To ensure ethnic homogeneity, all subjects, their parents and grandparents originated from and were born in Ireland [Republic of Ireland or Northern Ireland], Scotland, Wales or England; all were white. Subjects were questioned about any craniofacial trauma or surgery and individuals who reported such events were excluded. There were 13 male patients with bipolar disorder [mean age 45.0 (SD 14.3), range 22–63 years], 61 male controls [mean age 40.7 (SD 10.7), range 23–64 years], 14 female patients with bipolar disorder [mean age 44.7 (SD 16.3), range 17–65 years] and 75 female controls [mean age 38.0 (SD 9.7), range 20–58 years]; there were no patient–control differences for either sex (*P* > 0.05 for all comparisons).

Comparisons with schizophrenia involved the subjects of our recent study (Hennessy et al., 2007). There were 37 male patients with schizophrenia [mean age 46.5 (SD 12.4), range 23–64 years], 58 male controls [mean age 42.7 (SD 9.7), range 23–64 years], 32 female patients with schizophrenia [mean age 49.4 (SD 10.3), range 24–64 years] and 34 female controls [mean age 45.8 (SD 7.5), range 24–58 years]. Patients with bipolar disorder and schizophrenia, together with control subjects, were recruited and assessed in an identical manner, in close temporal contiguity, by the same investigators over the course of a common study protocol; control subjects entered into comparisons with bipolar patients overlap with those entered into comparisons with schizophrenia patients, supplemented by additional controls.

### 2.2 3D Laser surface imaging

**Fig. 1:**
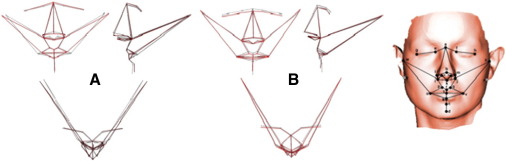
Visualisation of regression models for facial landmark analyses, separately for (A) male and (B) female bipolar patients *vs.* controls in (upper left) coronal, (upper right) sagittal and (bottom) axial planes. The Procrustes mean coordinates of facial landmarks for the pooled sample of subjects for each sex are joined by dashed black lines; the bipolar coordinates (the coordinates of a hypothetical bipolar patient of that sex lying along the bipolar–control discrimination axis) are joined by red lines that exaggerate the features of “patientness”, i.e. the difference of the patient from the sample mean, by a factor of approximately 5 to render them visible. Right inset: typical laser surface image (see Hennessy et al., 2007) showing the twenty-six 3D landmarks. Filled circles: the 24 landmarks used in landmark analysis. Open circles with crosses: the 2 landmarks used, in addition to the 24 landmarks, to calculate the pseudo-landmarks. Landmarks: a, soft tissue nasion; b, pronasale; c, sublabiale; d, pogonion; e/f, inner canthus; g/h, outer canthus; i/j, alar crest; k, subnasale; l/m, alare; n/o, columella breakpoint; p/q, christa philtrum; r, labiale superius; s, labiale inferius; t, stomion; u/v, cheilion; w/x, tragion; y/z, otobasion inferius.

Facial surfaces were recorded using a portable, hand-held Polhemus FastScan laser scanner (Hennessy et al., 2002, 2005). A typical surface, consisting of ∼ 80,000 points, has been shown previously in detail (Hennessy et al., 2007; see inset to Fig. 1).

### 2.3 Overview of analytical approach

In outline, the analytical approach was as follows: (i) identify manually a standard set of 24 3D landmarks over the facial surface; (ii) identify a much larger set of 1694 landmarks over the whole facial surface using an automated algorithm; (iii) apply geometric morphometrics to quantify and analyse differences in facial size and shape between groups; (iv) visualise the results of the resultant statistical models to extract biological meaning. It should be emphasised that the form of an object is a combination of size and shape, which are distinct constructs (Hennessy and Moss, 2001; Hennessy et al., 2005): a golf ball and a soccer ball have the same shape but different sizes; a balloon filled with a fixed quantity of water has a constant size that can be manipulated into different shapes. Additionally, the sexes differ intrinsically in facial size and shape (Hennessy et al., 2002; Evison et al., 2010), hence male and female subjects were analysed separately.

### 2.4 Landmark-based and pseudo-landmark approaches

Procedures for identifying 24 conventional 3D landmarks (Farkas, 1994) on these surfaces were as described previously in detail (Hennessy et al., 2004, 2005, 2007; see inset to Fig. 1) and carried out by a single investigator, blind to diagnostic category.

Procedures for specifying pseudo-landmarks [i.e. interpolated landmarks] over the whole facial surface were as described previously in detail (Hennessy et al., 2005, 2007). In outline this involves: 1) fitting each high resolution facial surface to a low-resolution template by means of thin plate splines and 26 control points — the 24 landmarks described below and an extra pair to improve accuracy; 2) locating a matching point on the high resolution surface for each point on the template. On this basis, 1694 pseudo-landmarks distributed over the entire facial surface were selected to provide sufficient resolution for visualisation of facial features.

The margins of the low-resolution template are set to extend over less of the facial surface than the high resolution images (Hennessy et al., 2005). Initial visualisations of all discrimination models [see below] are carefully checked to ensure that their behaviour at these margins is anatomically plausible. In the case of the female bipolar–control discrimination model, the surface at the superior margin of the forehead was observed to flex in an anatomically anomalous manner; this indicated that some facial scans in the female bipolar group did not extend sufficiently at the forehead. The low-resolution template was therefore edited to lower the facial margin, the data were reanalysed in all groups and no such anomalous behaviour was found. Thus, to allow valid comparisons between patients with bipolar disorder and those with schizophrenia, our previous analyses involving the schizophrenia group (Hennessy et al., 2007) were repeated here following the same editing.

### 2.5 Geometric morphometrics

As described previously (Hennessy et al., 2004, 2005, 2007), facial shape and size were analysed separately by scaling the original landmark sets to unit size as measured by centroid size, which quantifies the dispersion of the configuration of the landmark set as the square root of the sum of squared Euclidean distances of landmarks from their centre (Dryden and Mardia, 1998).

Covariance of facial shape with diagnostic category was analysed using geometric morphometrics, which allows shape covariance to be tested numerically and expressed visually (Hennessy et al., 2004, 2005, 2007). In outline, sets of scaled landmark coordinates were aligned with a registration algorithm (Generalised Procrustes Analysis) that has appropriate statistical properties (Rohlf, 1999). Transformed landmark residuals were analysed by principal component [PC] analysis to compute the major elements in shape variability within the sample and PCs with eigenvalues greater than the mean value, a standard selection criterion (Mardia et al., 1979), were selected for testing and modelling; this enables most of the shape variance to be captured in a modest number of PCs which is particularly advantageous for the pseudo-landmark sets, whose shape space has high dimensionality (Hammond et al., 2004).

### 2.6 Shape analysis and visualisation

For the 24 landmarks sets only, overall shape difference between groups and associated interaction terms were analysed using Procrustes ANOVA, as described previously (Klingenberg and McIntyre, 1998; Hennessy et al., 2004, 2005, 2007); comparable techniques for analysis of facial surfaces have yet to be developed. Subsequent analyses address the anatomical basis of shape difference and discrimination.

Initially, Goodall's test, a test of *overall* shape difference between two groups (Rohlf, 2000), was applied; this analysis composites all aspects of facial shape evaluated, both dysmorphic and non-dysmorphic, into a single metric and addresses whether an overall difference exists between the groups. Hotelling's *T*^2^ test, a parametric multivariate test of overall group discrimination, was then applied (Hennessy et al., 2005, 2007); this analysis similarly composites all aspects of facial shape evaluated, both dysmorphic and non-dysmorphic, into a single metric and addresses whether the groups can be discriminated, even if overall shape difference between them is small. Logistic regression was then carried out, with diagnostic category as the dependent variable and PCs as independent variables, to establish which individual aspect(s) of facial shape discriminate by diagnosis; both full regression models [including non-significant PCs] and parsimonious regression models [including only individually significant PCs] can be generated, with *R*^2^ values calculated using the ordinary least squares method. As in our previous reports (Hennessy et al., 2004, 2005, 2007), anatomical interpretations derive from parsimonious regression models, with PCs included if individually significant at *P* < 0.1. All analyses were carried out separately for males and females.

Regression models were visualised by multivariate regression of significant shape PCs onto the predicted values for the diagnosis variable (Klingenberg and Monteiro, 2005). The β coefficients of the model, which have the units of shape/diagnosis, were visualised by adding appropriately weighted eigenvectors to the Procrustes mean (O'Higgins, 2000), allowing the mean face to be morphed along the patient–control discrimination axis. The morphing is displayed dynamically, with multiple views to visualise the anatomy underlying statistical shape change.

Additionally, numerical information for the facial surface is colour coded to aid understanding (Hennessy et al., 2005, 2007): (a) change in surface vector area is coded as red [expanded] or blue [contracted]; (b) direction of displacement of surface is coded as red [angled outwards] or blue [angled inwards]; (c) length of displacement vector, in any direction, is coded as shades of red [darker shades code greater vector length].

## 3 Results

### 3.1 Facial landmark analysis: bipolar disorder *vs.* controls

Using the set of 24 biologically homologous landmarks, there was no difference in centroid size between bipolar patients and controls, for either males or females.

Procrustes ANOVA indicated overall facial shape to differ between bipolar patients and controls [effect of diagnosis, *P* < 0.001] and, as expected, between males and females [effect of sex, *P* < 0.001]. The absence of any diagnosis × sex interaction [*P* = 0.6] indicated that the difference in overall facial shape between bipolar patients and controls was generally similar in males and females.

Among males 14 PCs, describing 87.2% of total shape variance, were selected. Goodall's test of overall shape difference was significant [*P *< 0.05] and Hotelling's test of overall shape discrimination was non-significant [*P *= 0.2]. The parsimonious regression model [*R*^2^ = 18%] indicated the following individual PCs to distinguish male bipolar patients from controls: PC3: *β* = 238.3, SE*β* = 125.9, *P *= 0.06; PC4: *β* = − 394.2, SE*β* = 207.5, *P *= 0.06; PC5: *β* = 434.8, SE*β* = 189.7, *P *= 0.02. This regression model is visualised in Fig. 1a, where the mean male landmarks are shown, together with their locations along the bipolar patient–control discrimination axis that exaggerate the features of “patientness”, i.e. the difference of the patient from the sample mean, by a factor of approximately 5 to render them visible. The main features that discriminate male bipolar patients from controls were: the nose is turned down and lengthened; the mouth is narrow; the chin is set backwards and raised; the face is wider at tragion.

Among females 14 PCs, describing 86.6% of total shape variance, were selected. Both Goodall's test [*P *= 0.08] and Hotelling's test [*P *= 0.07] were marginal. The parsimonious regression model [*R*^2^ = 22%] indicated the following individual PCs to independently distinguish female bipolar patients from controls: PC1: *β* = − 217.8, SE*β* = 108.0, *P *= 0.04; PC5: *β* = − 474.0, SE*β* = 214.7, *P *= 0.03; PC6: *β* = − 483.1, SE*β* = 227.4, *P *= 0.03; PC8: *β* = − 478.1, SE*β* = 241.8, *P *= 0.05. This regression model is visualised in Fig. 1b, in the same manner as described above for males. The main features that discriminate female bipolar patients from controls were: the nose is turned up, wider at the base and shorter; the corners of the mouth are set forward with reduced mouth width; the chin is set higher and forward; the eyes are narrower; the face is wider at tragion and lengthened along the anterior–posterior axis.

### 3.2 Facial surface analysis: bipolar disorder *vs.* controls

Centroid size did not differ between male bipolar patients [2645.5 (SD 91.7) mm] and male controls [2656.2 (SD 80.6) mm; − 0.40%, *P *= 0.7] but was marginally larger in female bipolar patients [2484.3 (SD 61.3) mm] than in female controls [2443.3 (SD 74.7) mm; + 1.7%, *P *= 0.06]. All subsequent analyses of shape are independent of centroid size.

**Fig. 2:**
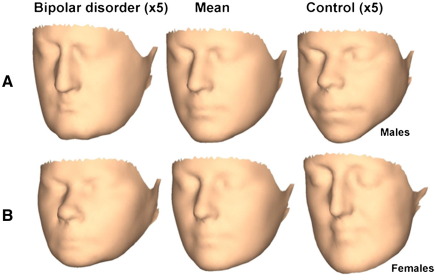
Visualisation of regression models for facial surface analyses, separately for (A) male and (B) female bipolar patients *vs.* controls. Procrustes mean shape is shown displaced equally in each direction along the bipolar–control discrimination axis. The displacement positions from mean facial shape (middle column) are exaggerated approximately 5-fold. This is equivalent to the patient (left column) — control (right column) dimorphism exaggerated approximately 10-fold.

**Fig. 3:**
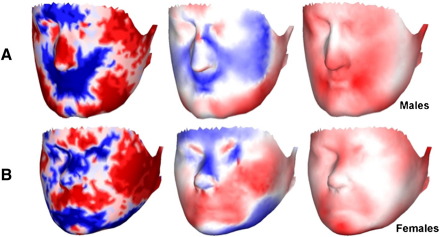
Visualisation of displacement vectors for each point on the surface, separately for (A) male and (B) female bipolar patients *vs.* controls. Left column: change in surface area coded as red [expanded in bipolar disorder relative to controls] or blue [contracted in bipolar disorder relative to controls]; data are coded as 33% percentiles, with darker colours indicating upper percentiles. Centre column: change in angle of displacement vector to surface normal coded as red [angled outward in bipolar disorder relative to controls] or blue [angled inward in bipolar disorder relative to controls]; darker colours code smaller angle to the normal, i.e. displacement vector more perpendicular to the surface. Right column: length of displacement vector, in any direction, coded as red; darker colour codes greater vector length, i.e. greater difference between bipolar disorder and controls.

Among males 12 PCs, describing 84.7% of total shape variance, were selected. Goodall's test was non-significant [*P *= 0.3] while Hotelling's test was marginal [*P *= 0.1]. The parsimonious regression model [*R*^2^ = 14%] indicated the following individual PCs to distinguish male bipolar patients from controls: PC5: *β* = − 4316.1, SE*β* = 2177.0, *P *= 0.05; PC6: *β* = 5021.1, SE*β* = 2528.0, *P *= 0.05; PC8: *β* = 5972.1, SE*β* = 2958.3, *P *= 0.04. This regression model is visualised in Fig. 2a, where the mean male face is shown, together with the facial surface at positions along the bipolar patient–control discriminating axis that exaggerate the features of “patientness” and “controlness” from the sample mean by a factor of approximately 5 to render them visible. In Fig. 3a, the mean face is colour coded to highlight topographically those geometric features that distinguish male bipolar patients from male controls.

In overall terms, the male bipolar patient face is laterally broad, lengthened anterio-posteriorly and the mouth is set posteriorly. Considered in more detail, the male bipolar patient face has the following features: the nose is turned down, lengthened and narrow; the mouth is narrow and set posteriorly; the chin is set forward; the mandible is wide; the cheeks are displaced inwards; the eyes are narrower; the face is wider at tragion. These surface findings elaborate those based on landmark data.

Among females 14 PCs, describing 87.1% of total shape variance, were selected. Goodall's test was non-significant [*P *= 0.3] while Hotelling's test was marginal [*P *= 0.1]. The parsimonious regression model [*R*^2^ = 8%] indicated the following individual PCs to distinguish female bipolar patients from controls: PC8: *β* = − 3810.3, SE*β* = 2506.5, *P *= 0.1; PC12: *β* = 7770.7, SE*β* = 3643.0, *P *= 0.03. This regression model is visualised in Fig. 2b, in the same manner as described above for males. In Fig. 3b, the mean face is colour coded to highlight topographically those geometric features that distinguish female bipolar patients from female controls.

In overall terms, the female bipolar patient face is vertically short, laterally broad and the mouth is set anteriorly. Considered in more detail, the female bipolar patient face has the following features: the nose is turned up, wider at the base, shorter with a recessed nasal bridge; the mouth is wider and set forward, with thinner lips; the chin is set higher and forward; the mandible is displaced upwards; the cheeks are displaced outwards; the eyes are narrower; the face is wider at tragion. These surface findings elaborate those based on landmark data.

### 3.3 Facial landmark and surface analyses: schizophrenia *vs.* controls

On repeating our initial analyses on the schizophrenia group (Hennessy et al., 2007) following the same template editing applied here to the bipolar group [see Section 2. Materials and methods], the parsimonious regression models were essentially unaltered.

### 3.4 Facial landmark analysis: bipolar disorder *vs.* schizophrenia

Using the set of 24 biologically homologous landmarks, there were no differences in centroid size between patients with bipolar disorder and those with schizophrenia, for either males or females.

Procrustes ANOVA indicated overall facial shape not to differ between bipolar and schizophrenia patients [no effect of diagnosis, *P* = 0.9] but, as expected, to differ between males and females [effect of sex, *P* < 0.001; no diagnosis × sex interaction, *P* = 0.4].

Among males 10 PCs, describing 83.2% of total shape variance, were selected. Neither Goodall's test [*P *= 0.6] nor Hotelling's test [*P *= 0.7] was significant. The parsimonious regression model [*R*^2^ = 7%] indicated only one PC to be a marginal predictor: PC5: *β* = − 347.9, SE*β* = 191.5, *P *= 0.07; thus, no regression model is visualised.

Among females 13 PCs, describing 89.4% of total shape variance, were selected. Neither Goodall's test [*P *= 0.7] nor Hotelling's test [*P *= 0.2] was significant. The parsimonious regression model [*R*^2^ = 9%] indicated only one PC to be a marginal predictor: PC8: *β* = 476.2, SE*β* = 240.2, *P *= 0.05; thus, no regression model is visualised.

### 3.5 Facial surface analysis: bipolar disorder *vs.* schizophrenia

Centroid size did not differ significantly between male bipolar patients [2645.5 (SD 91.7) mm] and male schizophrenia patients [2661.4 (SD 99.0) mm; − 0.6%, *P *= 0.6] or between female bipolar patients [2484.3 (SD 61.3) mm] and female schizophrenia patients [2507.8 (SD 99.5) mm; − 0.9%, *P *= 0.4]. All subsequent analyses of shape are independent of centroid size.

Among males 10 PCs, describing 82.8% of total shape variance, were selected. Neither Goodall's test [*P *= 0.7] nor Hotelling's test [*P *= 0.8] was significant. No PC entered a parsimonious regression model; thus, no regression model is visualised.

Among females 9 PCs, describing 82.3% of total shape variance, were selected. Neither Goodall's test [*P *= 0.6] nor Hotelling's test [*P *= 0.7] was significant. The parsimonious regression model [*R*^2^ = 7%] indicated only one PC to be a marginal predictor: PC8: *β* = − 5163.3, SE*β* = 2757.7, *P *= 0.06; thus, no regression model is visualised.

## 4 Discussion

This study applies 3D laser surface imaging and geometric morphometrics to capture, quantify and visualise, for the first time, subtle dysmorphologies of the face in bipolar disorder and compare them systematically with those evident in schizophrenia.

Regarding facial size, a slight increase (+ 1.7%) was found in female but not in male (− 0.4%) bipolar patients over the whole facial surface; no difference was found using facial landmark analysis. These findings are similar to those that we have reported previously in schizophrenia (+ 1.8% in female and + 0.03% in male patients over the whole facial surface; Hennessy et al., 2007) and may be associated with widening of the posterior skull base, as considered further below.

Regarding facial shape, while analysis over the whole facial surface in terms of pseudo-landmarks elaborated that based on landmarks, dysmorphology appeared more robust using facial landmark analysis. The whole facial surface is likely to involve both dysmorphic and non-dysmorphic regions, while the facial landmark set involves more circumscribed, primarily frontonasal regions that appear to be a particular focus for dysmorphology in bipolar disorder [and in schizophrenia (see Hennessy et al., 2007)]. Additionally, landmarks record the locations of anatomical points on the facial surface that are determined both by soft tissue and by underlying cartilage or bone, while pseudo-landmarks over the whole facial surface record primarily soft tissue. Though there may be subtle differences in dysmorphology on comparing landmark-based and whole surface analyses, for example in relation to some aspects of the mouth, these analytical approaches trade off anatomical specificity for breadth of capture and it is important to note the general convergence between them.

In terms of difference in overall shape on Procrustes ANOVA, both male and female bipolar patients evidenced dysmorphology, the extent of which did not differ between the sexes. In terms of topographical shape discrimination on 3D regression analysis, dysmorphology shared by male and female bipolar patients, particularly by landmark analysis, can be summarised as follows: overall facial widening; increased width of the nose; narrowing of the mouth; upward displacement of the chin. Shared regions of dysmorphology that evidence sexually dimorphic aspects can be summarised as follows: nose length is longer in male but shorter in female patients; the lips are thick in male patients but thin in female patients; tragion is displaced downwards and forwards in male patients but upwards and backwards in female patients.

We were unable to identify any material differences in total morphology between patients with bipolar disorder and those with schizophrenia, using either landmark-based or whole facial surface analyses. Among males, the 3D topography of dysmorphology in bipolar disorder [see Fig. 2 here] *vis-à-vis* schizophrenia [see Fig. 2 in our previous publication (Hennessy et al., 2007)] indicated more similarities than differences across these diagnostic groups. Dysmorphologies in males with bipolar disorder and schizophrenia are similar in terms of overall widening and vertical shortening of the face, down-turned nose and mouth narrowed and set backwards; there appeared to be some minor differences in terms of the cheeks which are displaced inwards in bipolar disorder but outwards in schizophrenia, the chin which is displaced forwards in bipolar disorder but backwards in schizophrenia and the jaws which are wide in bipolar disorder but narrow in schizophrenia. Among females, these same Figures indicate that dysmorphologies in bipolar disorder and schizophrenia are similar in terms of overall widening and vertical shortening of the face, outward displacement of the cheeks, outward and upward displacement of the jaw and upward displacement of the chin; there appeared to be some minor differences around the mouth, chin and nose tip which are displaced forward in bipolar disorder but backward in schizophrenia.

Before these findings can be interpreted biologically, it is necessary to consider methodological issues. As discussed previously in detail (Hennessy et al., 2007), patients and controls were well matched for age and sex and were drawn from the same rural region of substantive ethnic and socioeconomic homogeneity (Scully et al., 2004; Baldwin et al., 2005); thus, demographic, ethic and socioeconomic factors are unlikely to be prominent. Cavan-Monaghan Mental Health Service provides home-based care for acute illness as an alternative to admission, together with outpatient clinics, day hospital and day centre services (Baldwin et al., 2005); thus, nosocomial factors are unlikely to be operating. While the number of patients with bipolar disorder is smaller than for schizophrenia, this is offset by a large population of well matched control subjects drawn from individual and community group volunteers in the same catchment area. However, though we could detect differences between bipolar patients and controls, we cannot exclude that reduced statistical power may have contributed to failure to identify more prominent differences between bipolar patients and those with schizophrenia.

Another factor to be considered is a putative effect on facial shape of either weight loss due to poor self care or weight gain associated with antipsychotics or mood stabilisers (van Os and Kapur, 2009). Such a confound is unlikely for several reasons: (i) change in weight would be expected to affect primarily facial size, but this was altered minimally in females and not at all in males; (ii) differences in facial shape were topographically specific, with some regions expanded but others contracted, in a manner inconsistent with any overall effect of weight; (iii) the 24 landmark analysis would be expected to be considerably less sensitive to any such confounding, yet this evidenced an overall effect of diagnosis congruent to, though less detailed than, analyses on a “whole face” basis. To further exclude such an artefact, we analysed in females a subset of the face that excluded those maxillary and mandibular regions [i.e. “cheeks and jowls”] whose shape might be expected to particularly reflect weight change; on excluding these regions, patient–control differences endured. However, as body mass index data were not available in this study, future studies should include this variable in analyses.

Additionally, brain imaging studies can be influenced by age-related phenomena such as cerebral atrophy. In the present 3D craniofacial surface imaging studies, comparison groups were well matched for age. Facial size increases over childhood to reach mature values by the mid-teens, with minor variation thereafter; overall facial shape is determined considerably earlier, with only one among multiple components of facial shape continuing to evolve but reaching a plateau by the late teens (Hennessy and Moss, 2001; Evison et al., 2010).

Subject to these caveats, we report that patients with bipolar disorder are characterised by dysmorphology of frontonasal and adjacent facial areas. We have previously considered *in extensio* (Hennessy et al., 2007) the primarily midline process of normal anterior face and brain growth, narrowing of the anterior mid-facial region, primary palate formation, dissociation of cranial base width from anterior facial and cerebral changes, and more rapid forward growth of the face than of the brain (Waddington et al., 1999a,b; Diewert and Lozanoff, 1993a,b; Diewert et al., 1993; Cohen et al., 1993; Lieberman et al., 2000a,b) that we find here to be disrupted in bipolar disorder. That dysmorphology in bipolar disorder is more similar to than different from that found in schizophrenia using identical methods (Hennessy et al., 2007) implies some common pathobiology to dysmorphogenesis. These frontonasal and adjacent facial areas are related embryologically to forebrain and anterior midline cerebral regions and function as a single developmental unit in terms of 3D gene expression domains (Diewert and Lozanoff, 1993a,b; Diewert et al., 1993; Schneider et al., 2001; Marcucio et al., 2005; Kjaer, 1995; Echevarria et al., 2005; Tapadia et al., 2005; Aoto et al., 2009). The dysmorphologies that characterise bipolar disorder and schizophrenia implicate events acting particularly over a time-frame that has extreme limits of gestational weeks 6 through 19 but suggest a common denominator of weeks 9/10 through 14/15 of gestation (Waddington et al., 1999a,b; Diewert and Lozanoff, 1993a,b; Diewert et al., 1993; Cohen et al., 1993; Bayer and Altman, 2005; Hennessy et al., 2007).

Furthermore, that facial dysmorphologies characterising bipolar disorder and schizophrenia are similar but not identical would be consistent with evidence that patients with bipolar disorder and schizophrenia manifest similar but not identical abnormalities in terms of structural brain pathology (McDonald et al., 2005; Maier et al., 2006; Arnone et al., 2009; Fornito et al., 2009) and cognitive dysfunction (Krabbendam et al., 2005; Schretlen et al., 2007; Hill et al., 2008). It will be a task for future studies to clarify specific relationships to other psychotic diagnoses such as schizoaffective disorder, to non-psychotic diagnoses such as major depressive disorder, to affected and unaffected first-degree relatives, and to psychopathology, cognitive impairment, neurological signs and structural brain pathology.

It has been suggested (Murray et al., 2004; van Os and Kapur, 2009) that bipolar disorder and schizophrenia reflect a shared genetic predisposition to psychosis but that additional genes and/or early insults, resulting in greater neurodevelopmental impairment, give rise to a schizophrenia rather than a bipolar phenotype, perhaps in a sexually dimorphic manner. While our findings give partial support to this proposition, such intimacy in the embryological relationship between the face and brain raises a variant possibility: that bipolar disorder and schizophrenia might reflect (i) similar insult(s) acting over slightly differing time-frames, (ii) slightly differing insult(s) acting over a similar time-frame, or (iii) some combination thereof.

While the present findings inform on but cannot in themselves resolve the conundrum of the “Kraepelinian dichotomy” (see Section 1. Introduction; Jablensky, 1999; Maier et al., 2006; Ivleva et al., 2008; Fischer and Carpenter, 2009), they indicate that these two diagnostic categories are characterised by some common frontonasal dysmorphology and, presumptively, by some common process of cerebral–facial dysmorphogenesis. Early developmental perturbation in bipolar disorder and its pathobiological relationship to schizophrenia may be illuminated by greater understanding of the genetic and epigenetic regulation of midline morphogenesis of the frontonasal prominence and the clinical correlates of dysmorphology.

## Role of funding source

This work was supported by the Stanley Medical Research Institute, The Wellcome Trust [086901/Z/08/Z], the National Biophotonics and Imaging Platform Ireland funded by the Irish Government's Programme for Research in Third Level Institutions, Cycle 4, National Development Plan 2007–2013, and Cavan-Monaghan Mental Health Service. These agencies had no further role in study design, collection, analysis and interpretation of data or in the decision to submit the paper for publication.

## Contributors

Robin Hennessy and John Waddington designed the study. Patrizia Baldwin and David Browne ascertained, assessed and scanned patients and controls. Robin Hennessy and Anthony Kinsella analysed the data. Robin Hennessy and John Waddington prepared the first draft of the manuscript. All authors contributed to and have approved the final manuscript and participated in the decision to submit for publication.

## Conflict of interest

All authors declare that they have no conflict of interest.
